# Every Saturday

**Published:** 1867-02

**Authors:** 


EVERY SATURDAY.—A journal of choice reading, selected from foreign current literature. Published by Ticknor & Fields, 124 Tremont street, Boston. It is of rare merit, and containing as it does the cream of foreign literature, cannot fail to be one of the most readable magazines.

